# Supplementary material for: Ampelisca eschrichtii Krøyer, 1842 (Ampeliscidae) of the Sakhalin Shelf in the Okhotsk Sea starve in summer and feast in winter
Source: PeerJ. 2018 Jun 22;6:e4841. doi: 10.7717/peerj.4841 (PMC6016533; doi:10.7717/peerj.4841)
Supplement: Table S1 [file peerj-06-4841-s001.docx]

| a | anaphase of mitosis |
| --- | --- |
| ac | accessory cells |
| bm | basal membrane |
| fc | cells of follicular epithelium |
| gz | germinal zone |
| lov | lumen of ovary |
| ly | lysed yolk |
| mc | mesodermal cells |
| og | oogonia |
| ow | ovarian wall |
| p | prophase of mitosis |
| pfc | cells of primary follicular epithelium |
| pog | primary oogonia |
| pvo | previtellogenic oocyte |
| rvo | resorption of vitellogenic oocyte by follicle cells |
| sfc | cells of secondary follicular epithelium |
| spc | spermatocytes |
| spf | spermatophore |
| spt | spermatids |
| spz | spermatozoa |
| t | telophase of mitosis |
| vo | vitellogenic oocyte |
