# Supplementary material for: Ampelisca eschrichtii Krøyer, 1842 (Ampeliscidae) of the Sakhalin Shelf in the Okhotsk Sea starve in summer and feast in winter
Source: PeerJ. 2018 Jun 22;6:e4841. doi: 10.7717/peerj.4841 (PMC6016533; doi:10.7717/peerj.4841)
Supplement: Table S2 [file peerj-06-4841-s002.docx]

| **VO Diameters** | ***F0*** | ***FII*** | ***FIV*** |
| --- | --- | --- | --- |
| **Maximum** | 0.57 | 0.32 | 0.38 |
| **Upper Quartile** | 0.52 | 0.27 | 0.35 |
| **Mean** | 0.37 | 0.20 | 0.30 |
| **Median** | 0.33 | 0.22 | 0.34 |
| **Lower Quartile** | 0.23 | 0.13 | 0.28 |
| **Minimum** | 0.00 | 0.11 | 0.13 |
| **N** | **18** | **13** | **7** |
